# Enhancing Diagnostic Accuracy by Utilizing Regional Cerebral Blood Flow (rCBF) as a Value-Added Variable to Dynamic Contrast-Enhanced Perfusion-Weighted Imaging for Differentiating Radionecrosis From Recurrent Brain Tumour

**DOI:** 10.7759/cureus.89006

**Published:** 2025-07-29

**Authors:** Ahmed Mohamedbaqer Easa, Mohanad Sahib, Hosein Ghanaati, Aidin Taghiloo, Madjid Shakoba

**Affiliations:** 1 Department of Radiology Technology, College of Health and Medical Technologies, Al-Ayen Iraqi University, Thi-Qar, IRQ; 2 Department of Radiological Techniques, College of Health and Medical Techniques, Al-Mustaqbal University, Babylon, IRQ; 3 Department of Radiology, Advanced Diagnostic and Interventional Radiology Research Center, Tehran University of Medical Sciences, Tehran, IRN; 4 Department of Medical Imaging, Arad Hospital, Tehran, IRN; 5 Department of Biostatistics, Advanced Diagnostic and Interventional Radiology Research Center, Tehran University of Medical Sciences, Tehran, IRN

**Keywords:** dynamic contrast sensitivity, perfusion-weighted imaging, radionecrosis, rcbf, recurrent brain tumour

## Abstract

Purpose

To enhance diagnostic accuracy by incorporating relative cerebral blood flow (rCBF) as an additional parameter to dynamic susceptibility contrast perfusion-weighted imaging (DSC-PWI) variables, thereby enabling the differentiation between radionecrosis and recurrent brain tumours.

Materials and methods

This study involved 54 patients who had previously undergone treatment for primary cerebral tumours. All participants received radiotherapy and underwent both conventional MRI and DSC-PWI. MRI scans were performed using a 1.5 T closed MRI scanner (MAGNETOM Aera, Siemens Healthineers, Erlangen, Germany), equipped with a 16-channel Head/Neck coil for image acquisition. Several perfusion variables were calculated from DSC-PWI, including rCBF, relative cerebral blood volume (rCBV), relative peak height (rPH), and relative percentage of signal intensity recovery (rPSR). Statistical analyses included ANOVA and receiver operating characteristic (ROC) curve analysis, with histopathological findings used as the reference standard for diagnostic correlation.

Results

A total of 38 patients (38/54, 70.4%) were diagnosed with tumour recurrence (TR), while 16 patients (16/54, 29.6%) were diagnosed with radionecrosis. The values of rCBF, rCBV, rPSR, and rPH were significantly higher (*P* < 0.05) in recurrent tumours compared to radionecrosis lesions. At a cutoff value of ≥1.42, rCBF demonstrated a sensitivity of 0.95 and a specificity of 0.94. The area under the curve (AUC) for rCBF peaked at approximately 0.97, surpassing other parameters: rCBV (0.948), rPSR (0.069), and rPH (0.871).

Conclusion

DSC-PWI is a diagnostic tool that helps differentiate between TR and radionecrosis using several variables, the most important of which is rCBF.

## Introduction

Treating aggressive brain tumours, such as glioblastoma, involves a combination of surgery, chemotherapy, and radiation therapy to enhance treatment effectiveness. However, these therapies may increase the risk of radionecrosis [1,2]. Differentiating between tumour recurrence (TR) and radionecrosis is challenging but crucial for timely intervention and accurate prognostic information. Various imaging techniques, such as MR spectroscopy, PET, SPECT, and dynamic susceptibility contrast perfusion-weighted imaging (DSC-PWI), have been proposed to distinguish between TR and radionecrosis [3-5]. DSC-PWI is particularly useful in assessing the vascular properties of lesions that show enhancement after treatment by measuring blood volume, blood flow, and vessel wall permeability [6-8]. However, the lack of standardized post-processing procedures in DSC-PWI studies has led to inconsistencies in defining threshold values for identifying post-treatment changes. Consequently, the reported sensitivity and specificity of these assessments vary [9,10]. The DSC-PWI method involves injecting a contrast agent into the bloodstream to track signal strength decreases using T2*-weighted images [7]. This helps assess various hemodynamic properties, including relative cerebral blood flow (rCBF), relative cerebral blood volume (rCBV), relative percentage of signal intensity recovery (rPSR), and relative peak height (rPH). While some studies have explored rCBF variation using DSC, they often lack details on its sensitivity, specificity, and diagnostic accuracy. Most research in this area focuses on the rCBV parameter [11,12]. Therefore, the objective of our study is to emphasize the diagnostic significance of the rCBF variable, along with other perfusion metrics.

## Materials and methods

Patients

This prospective study enrolled 54 patients out of 102; 48 patients were excluded due to missing biopsy data. These patients had previously undergone neurosurgery and received adjuvant radiotherapy for brain tumours. They were referred to the Imaging Center between February 2023 and January 2025 due to suspected TR or radionecrosis.

Imaging protocol

MRI scans were conducted using a 1.5 T closed MRI scanner manufactured by Siemens, specifically the MAGNETOM Aera model from Germany, which utilized a 16-channel Head/Neck coil for image acquisition. The imaging protocol included the following sequences: a quick three-plane localizer sequence for localization and planning (less than 25 seconds); axial and sagittal T1-weighted spin echo sequences (TR: 600 ms, TE: 18 ms); T2-weighted axial, sagittal, and coronal turbo spin echo sequences (TR: 4000 ms, TE: 120 ms); an axial T2-weighted fluid-attenuated inversion recovery (FLAIR) sequence (TR: 9000 ms, TE: 110 ms, TI: 2500 ms); a DWI sequence acquired axially with two b-values (0 and 1000 s/mm²); dynamic susceptibility contrast (DSC) perfusion gradient-echo echoplanar imaging with the gadolinium-based contrast agent Dotarem, administered at 0.2 mL/kg (0.1 mmol/kg) as a bolus injection; and a post-contrast T1-weighted spin echo sequence (TR: 570 ms, TE: 10 ms).

Processing and analyzing the images

**Figure 1:**
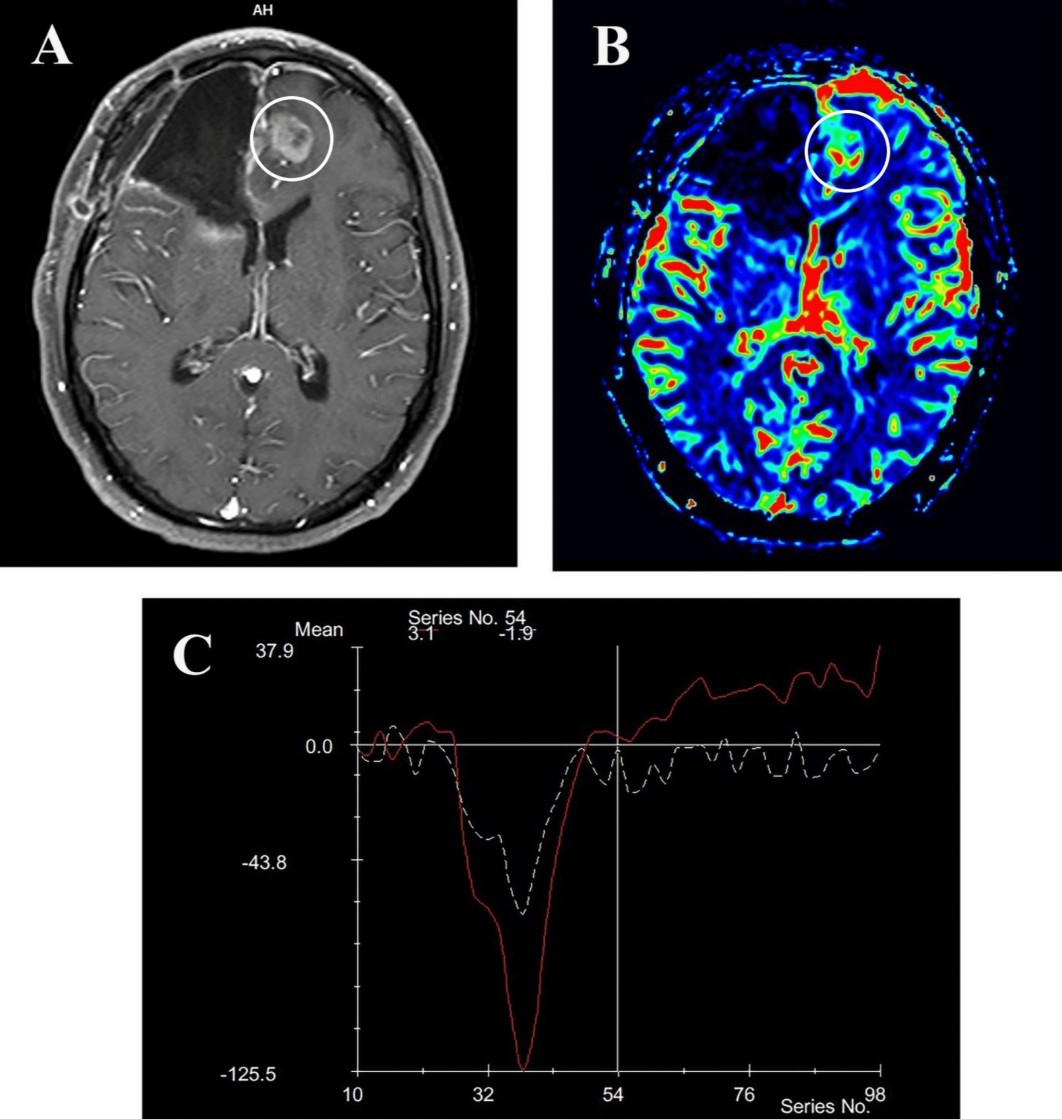
Shows a 46-year-old male patient with a history of GBM surgically removed from the right frontal lobe, followed by chemotherapy and radiotherapy. Histopathology confirmed the excised GBM as a recurrence. (A) Post-contrast T1-weighted axial MRI reveals a new enhancing mass in the left frontal paramidline lobe, surrounded by vasogenic edema. (B) The CBF color map shows higher perfusion compared to the opposite NAWM, with rCBF measured at 6.83. (C) The T2*-weighted signal intensity–time curve displays the lesion’s rPH = 4.28 and rPSR = 0.64, compared to the contralateral NAWM, represented by the yellow dashed line. GBM: Glioblastoma Multiforme; CBF: Cerebral Blood Flow; rCBF: Relative Cerebral Blood Flow; rPH: Relative Peak Height; rPSR: Relative Percentage of Signal Intensity Recovery; NAWM: Normal-Appearing White Matter.

**Figure 2:**
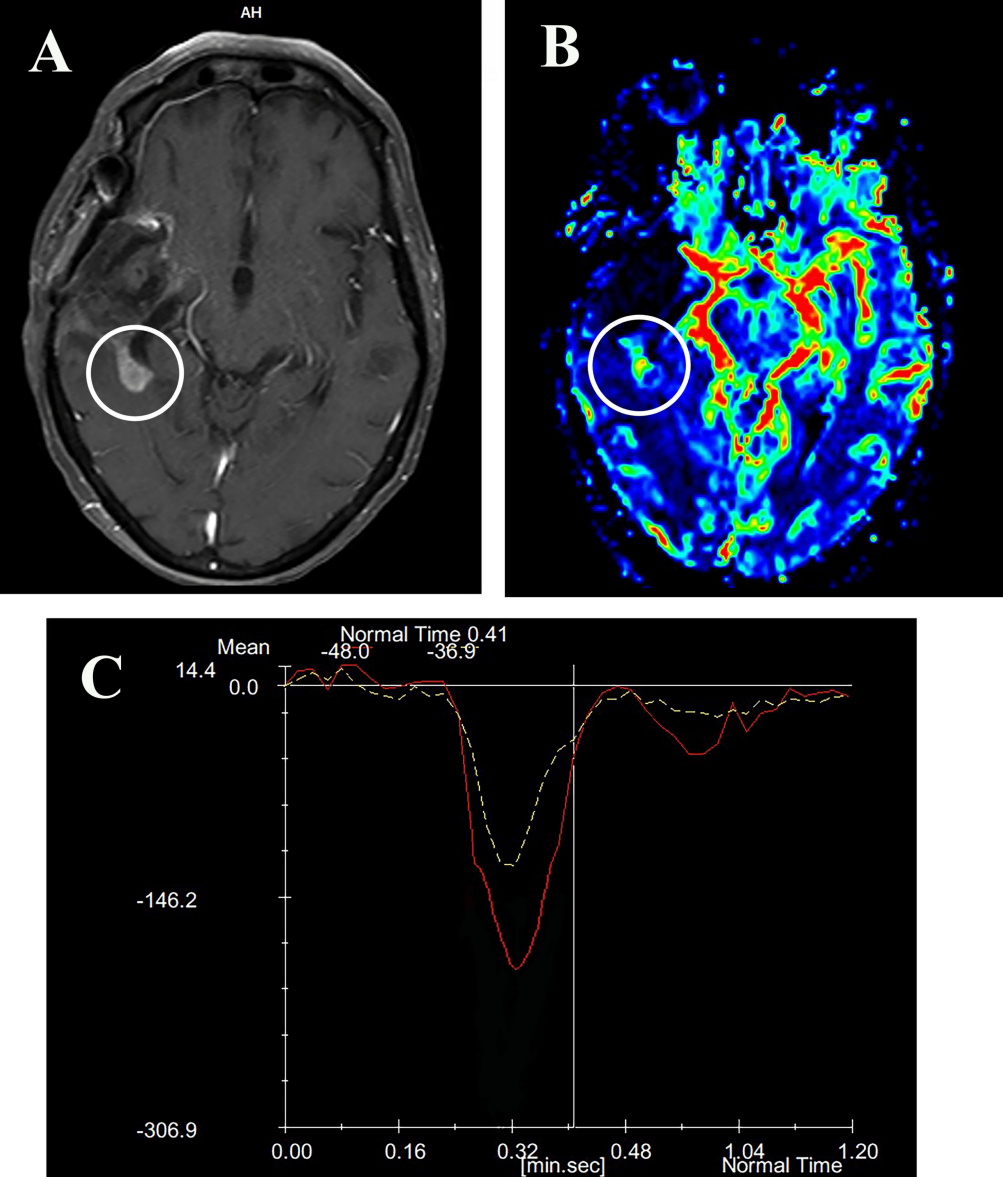
Shows a 63-year-old female patient with a history of GBM surgical removal. Following surgery, chemotherapy, and radiotherapy, histopathological analysis revealed radiation necrosis in the excised GBM. (A) Post-contrast T1-weighted axial MRI showing a new enhancing mass in the posterior temporal region, accompanied by edema. (B) CBF color map demonstrating higher perfusion (relative CBF (rCBF) = 0.92) compared to the NAWM on the opposite side. (C) T2*-weighted signal intensity-time curve showing the lesion’s rPH = 1.23 and rPSR = 0.92, relative to the contralateral NAWM (indicated by the yellow dashed line). GBM: Glioblastoma Multiforme; CBF: Cerebral Blood Flow; rCBF: Relative Cerebral Blood Flow; rPH: Relative Peak Height; rPSR: Relative Percentage of Signal Intensity Recovery; NAWM: Normal-Appearing White Matter.

The DSC and conventional MRI data were transferred to an offline workstation for further analysis. T2*-weighted signal intensity-time curves were generated voxel by voxel. ROI analysis was performed for CBF measurements. Initially, a control ROI (CBF_NAWM_) was drawn in the normal-appearing white matter (NAWM) opposite the enhancing lesion. Subsequently, two to three circular ROIs (approximately 0.5 mm²) were placed within the enhancing lesion on the CBF map, specifically targeting regions with high visual CBF values while excluding cystic or necrotic areas, blood vessels, hemorrhage, and susceptibility artifacts. The most significant rCBF was calculated by dividing the CBF for the lesion (CBF_lesion_) by CBF_NAWM_. rCBV was calculated similarly to rCBF, as demonstrated in a patient with confirmed recurrent GBM showing elevated perfusion and contrast enhancement on MRI (Figure 1). Two variables, S_0_ (baseline signal intensity before contrast administration) and S_min _(minimum signal intensity at the peak of the contrast bolus), were derived from the signal intensity-time curve for both NAWM and the enhancing lesion. These values were then used to compute the relative peak height (rPH) using the provided equation: rPH = (S_0(lesion) _- S_min(lesion)_) / (S_0(NAWM)_ - S_min(NAWM)_) Similarly, the following equation was used to determine rPSR for all patients: rPSR = {(S_1(ROI)_ - S_min(ROI)_) / (S_0(ROI)_ - S_min(ROI)_)} / {(S_1(NAWM)_ - S_min(NAWM)_) / (S_0(NAWM)_ - S_min(NAWM)_)}, where S_1(ROI)_ represents the post-contrast T2*-weighted signal intensity of the ROI, and S_1(NAWM)_ represents that of the NAWM. The ROI was set to the highest signal within the lesion for image processing. Maximum values were calculated for rCBV, rPH, and rPSR, as they provide the most accurate measurements according to previous studies. In contrast, as illustrated in Figure 2, a case of radiation necrosis demonstrates markedly lower perfusion and subdued signal curve parameters, clearly distinguishing it from TR.

Statistical analysis

Statistical analyses were conducted using IBM SPSS Statistics (version 26). Quantitative data are expressed as means ± SD, while categorical data are presented as counts and percentages. The Kolmogorov-Smirnov test was used to assess the normality of continuous variables. For group comparisons, independent samples t-tests were used for normally distributed data, while the Mann-Whitney U test was applied for non-normally distributed data. Chi-square (χ²) tests were used for comparing categorical variables. Statistical significance was defined as p < 0.05.

Chi-square and F-values were estimated using the following approximation formulas:

χ^2^≈t^2^, F≈t^2^\χ^2^\approx t^2^\quad F\approx t^2^

Receiver operating characteristic (ROC) curve analysis was performed to evaluate the diagnostic performance of each perfusion variable. The following diagnostic metrics were calculated along with their confidence intervals: area under the curve (AUC), sensitivity, specificity, accuracy, positive predictive value (PPV), negative predictive value (NPV), positive likelihood ratio (PLR), and negative likelihood ratio (NLR).

## Results

In this study, 54 patients participated, with a mean age of 47.9 ± 13.6 years (range: 22 to 78 years). Among them, 33 patients were male (61.1%). Pathology results showed that 38 patients (70.4%) had TR, with different types and grades of brain tumours, including glioblastoma, diffuse astrocytoma, anaplastic astrocytoma, oligodendroglioma, anaplastic oligodendroglioma, pilocytic astrocytoma, ependymoma, and anaplastic ependymoma, with grades ranging from 1 to 4. Meanwhile, 16 patients (29.6%) were diagnosed with radionecrosis. Mean perfusion variables are provided in Table 1.

**Table 1:** Mean values of DSC-PWI variables among the patients. rCBF: relative cerebral blood flow; rCBV: relative cerebral blood volume; rPSR: relative percentage of signal intensity recovery; rPH: relative peak height; P-value: Probability value indicating statistical significance.; DSC-PWI: Dynamic susceptibility contrast perfusion-weighted imaging.

Variable	N	Minimum	Maximum	Mean	SD
rCBF	54	0.26	8.79	2.91	1.85
rCBV	54	0.28	9.02	2.73	1.74
rPSR	54	0.34	1	0.73	0.16
rPH	54	0.36	7.64	2.17	1.36

Among patients with TR, 26 out of 38 were male (68.4%), while in the radionecrosis group, 7 out of 16 were male (43.8%; P = 0.09). The mean age in the TR group was 46.2 ± 13.3 years, and in the radionecrosis group, it was 51.9 ± 13.8 years (p = 0.16). Significant differences in mean perfusion parameters were observed between the two groups, particularly in the mean maximum rCBF, which was 3.71 ± 1.57 for recurrence and 0.99 ± 0.63 for radionecrosis (p < 0.001). Additional comparisons are presented in Table 2.

**Table 2:** Comparison of mean, SD, test statistics, and p-values of DSC-PWI variables between tumour recurrence and radionecrosis groups. t-value: Student's t-test value; p-value: probability value; χ²: Chi-square statistics; F: F-test value (ANOVA); rCBF: relative cerebral blood flow; rCBV: relative cerebral blood volume; rPSR: relative percentage of signal intensity recovery; rPH: relative peak height; DSC-PWI: Dynamic susceptibility contrast perfusion-weighted imaging; TR: Tumour Recurrence.

Variable	Group	Mean	SD	t-value	p-value	χ²	F
rCBF	TR	3.71	1.57	8.64	<0.001	74.66	74.66
	Radionecrosis	0.99	0.63				
rCBV	TR	3.42	1.57	8.02	<0.001	64.32	64.32
	Radionecrosis	1.06	0.67				
rPSR	TR	0.66	0.13	-7.30	<0.001	53.29	53.29
	Radionecrosis	0.89	0.09				
rPH	TR	2.64	1.3	6.85	<0.001	46.92	46.92
	Radionecrosis	1.04	0.63				

**Figure 3:**
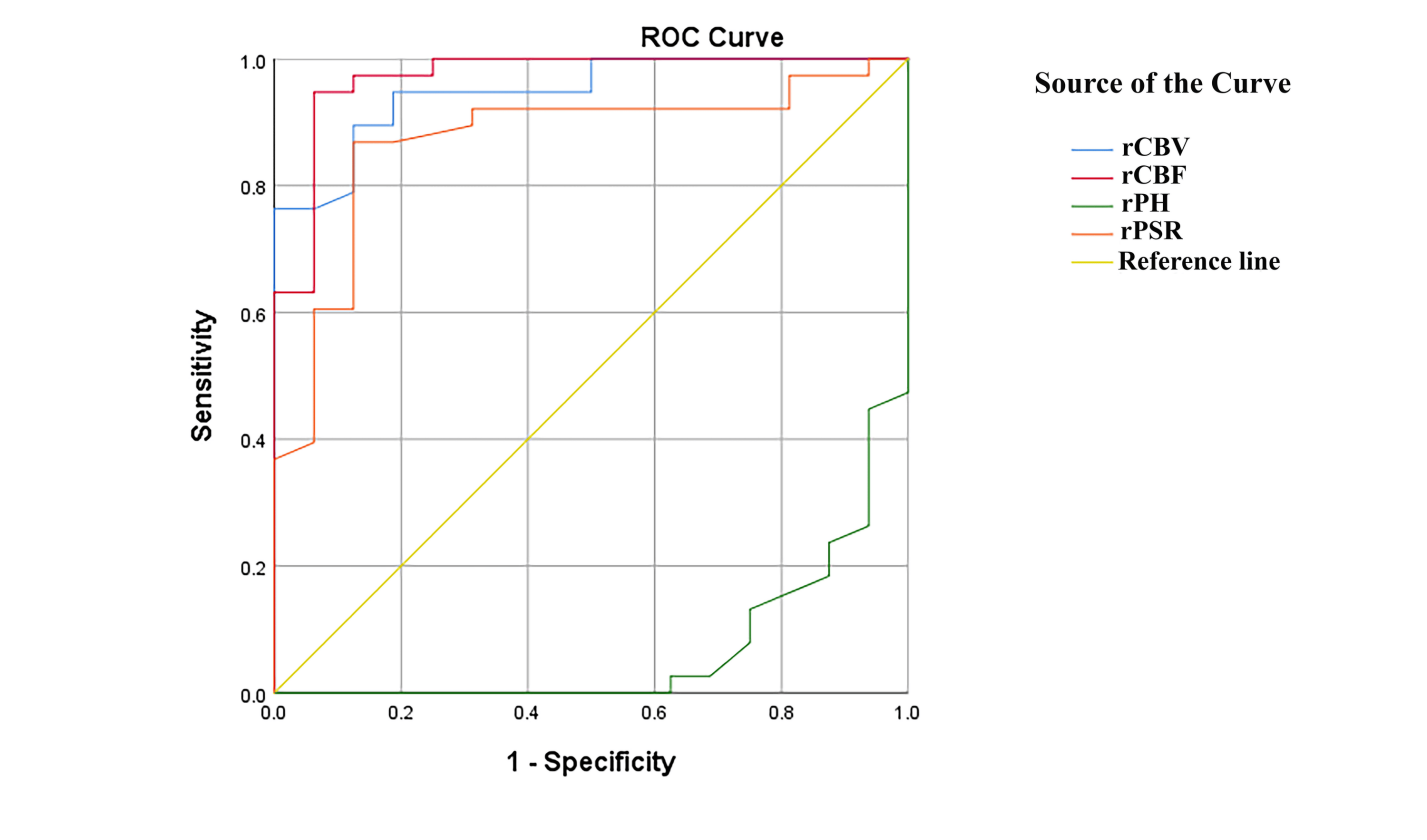
Shows the AUC values for the DSC-PWI variables. DSC-PWI: Dynamic susceptibility contrast perfusion-weighted imaging; AUC: Area under the curve.

For differentiating TR, ROC analysis was conducted, and rCBF showed the best performance, with an AUC of 0.97, as reported in Table 3 along with other variables. The results of the perfusion variables from DSC-PWI across various cutoff points showed that sensitivity, specificity, accuracy, PPV, NPV, PLR, and NLR were all significant, as shown in Table 4 and Figure 3. 

**Table 3:** Diagnostic accuracy of DSC-PWI variables based on ROC curve analysis and corresponding AUC values. AUC: Area under the curve; Std. error: Standard Error; t-value: Student's t-test value; χ²-value: Chi-square statistics; F-value: F-test value (ANOVA); rCBF: relative cerebral blood flow; rCBV: relative cerebral blood volume; rPSR: relative percentage of signal intensity recovery; rPH: relative peak height; P-value: Probability value indicating statistical significance; DSC-PWI: Dynamic susceptibility contrast perfusion-weighted imaging; 95% CI = 95 Percent Confidence Interval (Lower-Upper). Note: t-values were calculated using the formula: t=AUC−0.5SEt = \frac{{AUC - 0.5}}{{SE}} χ² and F-values estimated as: χ^2^≈t^2^,F≈t^2^ (for single predictor)χ² ≈ t^^2^,\quad F ≈ t^2^ \text{ (for single predictor)}

Test Variable	AUC	Std. Error	p-value	t-Value	χ² Value	F-value	95% CI (Lower-Upper)
rCBF	0.97	0.024	<0.001	19.58	383.9	383.9	0.923-1.000
rCBV	0.948	0.028	<0.001	16	256	256	0.894-1.000
rPSR	0.069	0.036	<0.001	-11.94	142.52	142.52	0.000-0.140
rPH	0.871	0.053	<0.001	7.01	49.14	49.14	0.767-0.975

**Table 4:** Diagnostic performance metrics including χ² and F values, with sensitivity, specificity, accuracy, PPV, NPV, PLR, and NLR. Cutoff: Cutoff value; Sens: Sensitivity; Spec: Specificity; Acc: Accuracy; PPV: Positive predictive value; NPV: Negative predictive value; PLR: Positive likelihood ratio; NLR: Negative likelihood ratio; t-value: Student’s t-test value; p-value: Probability value; χ²: Chi-square statistic; F: F-test value (ANOVA); rCBF: relative cerebral blood flow; rCBV: relative cerebral blood volume; rPSR: relative percentage of signal intensity recovery; rPH: relative peak height; DSC-PWI: Dynamic susceptibility contrast perfusion-weighted imaging.

Variable	Cutoff	Sens	Spec	Acc.	PPV	NPV	PLR	NLR	*t*-Value	*P*-Value	χ²	F
rCBF	≥1.42	0.95	0.94	0.94	0.97	0.88	15.16	0.06	8.64	<0.001	74.66	74.66
rCBV	≥1.39	0.89	0.88	0.89	0.94	0.78	7.16	0.13	8.02	<0.001	64.32	64.32
rPSR	≤0.81	0.87	0.75	0.83	0.89	0.71	3.47	0.17	-7.30	<0.001	53.29	53.29
rPH	≥1.50	0.87	0.88	0.87	0.94	0.74	6.94	0.15	6.85	<0.001	46.92	46.92

## Discussion

Further research is needed to differentiate radionecrosis from TR in clinical neuro-oncology. Accurate identification is crucial for treatment planning. Histopathological examination reveals distinct differences: radionecrosis shows fibrinoid necrosis and vascular injury, while recurrent tumours exhibit increased tumour vasculature density [11]. Biopsies carry risks of sampling errors [12]. Noninvasive alternatives like MR spectroscopy, PWI, DWI, and PET can assist in distinguishing between the two conditions.

rCBF and rCBV

Our research highlights the importance of rCBF as a very precise factor in DSC perfusion analysis, whereas the majority of studies concentrated on rCBV. In the group of patients with TR, the average rCBF was 3.71±1.57, which was notably higher than the necrosis group where it averaged 0.99±0.63 (P<0.001). There have been only a few studies on rCBF in DSC perfusion MRI, such as the one by Muto M et al., which also found significant rCBF values (P < 0.05). They observed higher rCBF in cases of TR compared to changes related to necrosis [11-13]. In addition, Shah AH et al. found a significant relationship between increased CBF and active tumour percentage (P = 0.0004). They also discovered an interaction between blood flow and tumour histological features [12-14]. Our study was unique in focusing on the rCBF variable, and it showed results with an accuracy of 0.94 and an AUC of 0.97 to differentiate between TR and RN. Research findings confirmed significantly higher mean rCBV values in the recurrence group (3.42±1.57) compared to the necrosis group (1.06±0.67) (P<0.001). Several studies, including Roques M et al., support the use of rCBV to differentiate between radionecrosis and TR, with lower rCBVmax (rCBV maximum) observed in radionecrosis compared to tumour progression [15]. A study by Elshafeey N et al. showed that CBV is effective in distinguishing pseudoprogression from progressive disease with 90.82% accuracy (AUC = 89.10%, sensitivity = 91.36%, specificity = 88.24%, P = 0.017) [16]. Steidl E et al. found that an optimized cutoff of 2.75 for corrected rCBVmax achieved 89% sensitivity and 86% specificity, while an optimized cutoff of 1.68 for uncorrected rCBVmax yielded 89% sensitivity but only 41% specificity [17]. Regarding the outcomes of our study, it demonstrated similar sensitivity (89%) and specificity (88%) as the previously mentioned studies.

rPH and rPSR

The study observed that the recurrence group had a mean rPH of 2.64±1.30, while the necrosis group had a mean rPH of 1.04±0.63. Accuracy, sensitivity, and specificity were 0.87, 0.87, and 0.88, respectively. rPH proved to be a more specific indicator than rPSR in differentiating between TR and radionecrosis, with a specificity of 88% compared to 75%. This finding aligns with the results reported by Barajas RF Jr et al. [18]. Furthermore, Young RJ et al. concluded that rPH was the most reliable predictor, achieving a sensitivity of 100% at a cutoff of 1.7 in their respective studies [19].

In our study, we used rPSR from T2*-weighted DSC MR imaging to estimate capillary leakiness, which helps distinguish between radionecrosis and recurrent brain tumours. The recurrence group had a mean rPSR of 0.66±0.13, while the necrosis group had a mean rPSR of 0.89±0.09. The accuracy, sensitivity, and specificity were 0.83, 0.87, and 0.75, respectively, with a cutoff point of ≤0.84. These findings align with other studies, such as Barajas RF Jr et al., where a cutoff value of 87.3% yielded a sensitivity of 78.26% and a specificity of 76.19% [18]. Additionally, Smitha KA et al. demonstrated that rPSR outperformed rCBV and rCBF values in distinguishing tumour grade [20]. Based on these findings, it is recommended to consider all DSC-PWI variables, with a particular focus on rCBF due to its significant diagnostic value in achieving high-quality results. However, a major limitation of this study is the lack of biopsy data for many patients, which led to their exclusion. In addition, the sample size is relatively small, with only 16 patients in the radionecrosis group.

## Conclusions

This study highlights the high diagnostic value of rCBF in DSC-PWI for distinguishing tumour recurrence from radionecrosis, demonstrating superior accuracy (AUC = 0.97) compared to traditional markers such as rCBV. Incorporating rCBF alongside rPH and rPSR enhances non-invasive assessment and reduces reliance on high-risk biopsies.

Clinically, these findings support the integration of rCBF into routine imaging protocols to guide treatment decisions more effectively. However, the study’s limitations, such as a small sample size and the exclusion of patients due to missing biopsy data, warrant cautious interpretation.

Future research should focus on larger, multicenter studies with standardized imaging and analysis protocols to validate rCBF cutoffs and improve diagnostic consistency. Overall, rCBF emerges as a key parameter in advancing diagnostic precision in neuro-oncology.

## References

[1] Zhang J, Wang Y, Wang Y (2022). Perfusion magnetic resonance imaging in the differentiation between glioma recurrence and pseudoprogression: a systematic review, meta-analysis and meta-regression. Quant Imaging Med Surg.

[2] Chuang MT, Liu YS, Tsai YS, Chen YC, Wang CK (2016). Differentiating radiation-induced necrosis from recurrent brain tumor using MR perfusion and spectroscopy: a meta-analysis. PLoS One.

[3] Detsky JS, Keith J, Conklin J (2017). Differentiating radiation necrosis from tumor progression in brain metastases treated with stereotactic radiotherapy: utility of intravoxel incoherent motion perfusion MRI and correlation with histopathology. J Neurooncol.

[4] Shah AH, Snelling B, Bregy A (2013). Discriminating radiation necrosis from tumor progression in gliomas: a systematic review what is the best imaging modality?. J Neurooncol.

[5] Filss CP, Cicone F, Shah NJ, Galldiks N, Langen KJ (2017). Amino acid PET and MR perfusion imaging in brain tumours. Clin Transl Imaging.

[6] Soliman HM, ElBeheiry AA, Abdel-Kerim AA (2018). Recurrent brain tumor versus radiation necrosis; can dynamic susceptibility contrast (DSC) perfusion magnetic resonance imaging differentiate?. Egypt J Radiol Nucl Med.

[7] Boxerman JL, Ellingson BM, Jeyapalan S (2017). Longitudinal DSC-MRI for distinguishing tumor recurrence from pseudoprogression in patients with a high-grade glioma. Am J Clin Oncol.

[8] Welker K, Boxerman J, Kalnin A, Kaufmann T, Shiroishi M, Wintermark M (2015). ASFNR recommendations for clinical performance of MR dynamic susceptibility contrast perfusion imaging of the brain. AJNR Am J Neuroradiol.

[9] Wan B, Wang S, Tu M, Wu B, Han P, Xu H (2017). The diagnostic performance of perfusion MRI for differentiating glioma recurrence from pseudoprogression: a meta-analysis. Medicine (Baltimore).

[10] Huang RY, Neagu MR, Reardon DA, Wen PY (2015). Pitfalls in the neuroimaging of glioblastoma in the era of antiangiogenic and immuno/targeted therapy - detecting illusive disease, defining response. Front Neurol.

[11] Muto M, Frauenfelder G, Senese R, Zeccolini F, Schena E, Giurazza F, Jäger HR (2018). Dynamic susceptibility contrast (DSC) perfusion MRI in differential diagnosis between radionecrosis and neoangiogenesis in cerebral metastases using rCBV, rCBF and K2. Radiol Med.

[12] Shah AH, Kuchakulla M, Ibrahim GM, Dadheech E, Komotar RJ, Gultekin SH, Ivan ME (2019). Utility of magnetic resonance perfusion imaging in quantifying active tumor fraction and radiation necrosis in recurrent intracranial tumors. World Neurosurg.

[13] Jahng GH, Li KL, Ostergaard L, Calamante F (2014). Perfusion magnetic resonance imaging: a comprehensive update on principles and techniques. Korean J Radiol.

[14] Castro-Giner F, Gkountela S, Donato C (2018). Cancer diagnosis using a liquid biopsy: challenges and expectations. Diagnostics (Basel).

[15] Roques M, Catalaa I, Raveneau M (2022). Assessment of the hypervascularized fraction of glioblastomas using a volume analysis of dynamic susceptibility contrast-enhanced MRI may help to identify pseudoprogression. PLoS One.

[16] Elshafeey N, Kotrotsou A, Hassan A (2019). Multicenter study demonstrates radiomic features derived from magnetic resonance perfusion images identify pseudoprogression in glioblastoma. Nat Commun.

[17] Steidl E, Müller M, Müller A, Herrlinger U, Hattingen E (2019). Longitudinal, leakage corrected and uncorrected rCBV during the first-line treatment of glioblastoma: a prospective study. J Neurooncol.

[18] Barajas RF Jr, Chang JS, Segal MR, Parsa AT, McDermott MW, Berger MS, Cha S (2009). Differentiation of recurrent glioblastoma multiforme from radiation necrosis after external beam radiation therapy with dynamic susceptibility-weighted contrast-enhanced perfusion MR imaging. Radiology.

[19] Young RJ, Gupta A, Shah AD (2013). MRI perfusion in determining pseudoprogression in patients with glioblastoma. Clin Imaging.

[20] Smitha KA, Gupta AK, Jayasree RS (2015). Relative percentage signal intensity recovery of perfusion metrics—an efficient tool for differentiating grades of glioma. Br J Radiol.

